# In Vitro Metabolism of Δ^8^‐, Δ^9^‐, and Δ^10^‐THC in Human Hepatocytes: Distinct Δ^10^‐THC Biotransformation and Implications for Drug Testing

**DOI:** 10.1002/dta.70101

**Published:** 2026-06-02

**Authors:** Robert Kronstrand, Henrik Green, Markus Loh, Fabian Rüttimann, Manuela Carla Monti

**Affiliations:** ^1^ Department of Biomedical and Clinical Science, Division of Clinical Chemistry and Pharmacology Linköping University Linköping Sweden; ^2^ Department of Forensic Genetics and Forensic Toxicology National Board of Forensic Medicine Linköping Sweden; ^3^ School of Life Sciences FHNW University of Applied Sciences and Arts Northwestern Switzerland Muttenz Switzerland; ^4^ Institute of Forensic Medicine, Department of Biomedical Engineering University of Basel Basel Switzerland

**Keywords:** cannabis, forensic toxicology, metabolism, semi‐synthetic cannabinoids, SSC

## Abstract

With its widespread recreational and increasing medical use, ∆^9^‐tetrahydrocannabinol (∆^9^‐THC), the main psychoactive compound in cannabis, is regularly subjected to drug testing in clinical and forensic toxicology. ∆^8^‐ and ∆^10^‐THC have recently entered unregulated drug markets. Their close structural similarity to ∆^9^‐THC poses various analytical challenges, with a high risk of misidentification and misinterpretation of bioanalytical data. This study investigated the in vitro metabolic fate of ∆^8^‐ and ∆^10^‐THC in comparison to ∆^9^‐THC using human hepatocytes and high‐performance liquid chromatography coupled to high‐resolution time‐of‐flight analysis (HPLC‐QToF). A comparable metabolism was observed for ∆^8^‐THC and ∆^9^‐THC, with the formation of the monohydroxy and carboxy metabolites and their glucuronides. In contrast, ∆^10^‐THC was found to be extensively glucuronidated (forming ∆^10^‐THC‐glucuronide) and monohydroxylated, with only minor formation of a carboxy metabolite. Structural considerations led to the hypothesis that ∆^10^‐THC is predominantly hydroxylated at a different site than ∆^8^‐THC and ∆^9^‐THC. THC isomers should be considered in cannabis drug testing. The differing metabolism of ∆^10^‐THC exacerbates the risk of misinterpretation of analytical results.

## Graphical abstract

Δ^8^‐THC and Δ^9^‐THC showed comparable metabolic profiles. In contrast, Δ^10^‐THC underwent extensive glucuronidation and hydroxylation, with only minor formation of a carboxy metabolite, indicating a markedly different metabolic pathway. These differences may increase the risk of misidentification and misinterpretation in cannabis drug testing.

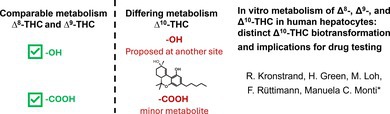

## 1 Introduction

∆^9^‐Tetrahydrocannabinol (∆^9^‐THC), the main psychoactive constituent in cannabis, has been a key analyte for cannabis‐related analyses in forensic toxicology (e.g., driving under the influence [DUI]) and workplace drug testing [1, 2]. However, with the emergence of closely related isomers and homologues of ∆^9^‐THC, a diversity of new cannabinoid compounds, so‐called semi‐synthetic cannabinoids (SSCs), is now available on unregulated and recreational drug markets. The term “semi‐synthetic” originates from the fact that several of these THC analogues can be synthesized using phytocannabinoids, such as the nonintoxicating phytocannabinoid cannabidiol (CBD), as starting materials. This approach is likely inspired by the increased production of CBD‐rich cannabis (hemp) over the last years following legislative changes in Europe and the United States [3, 4].

**FIGURE 1:**
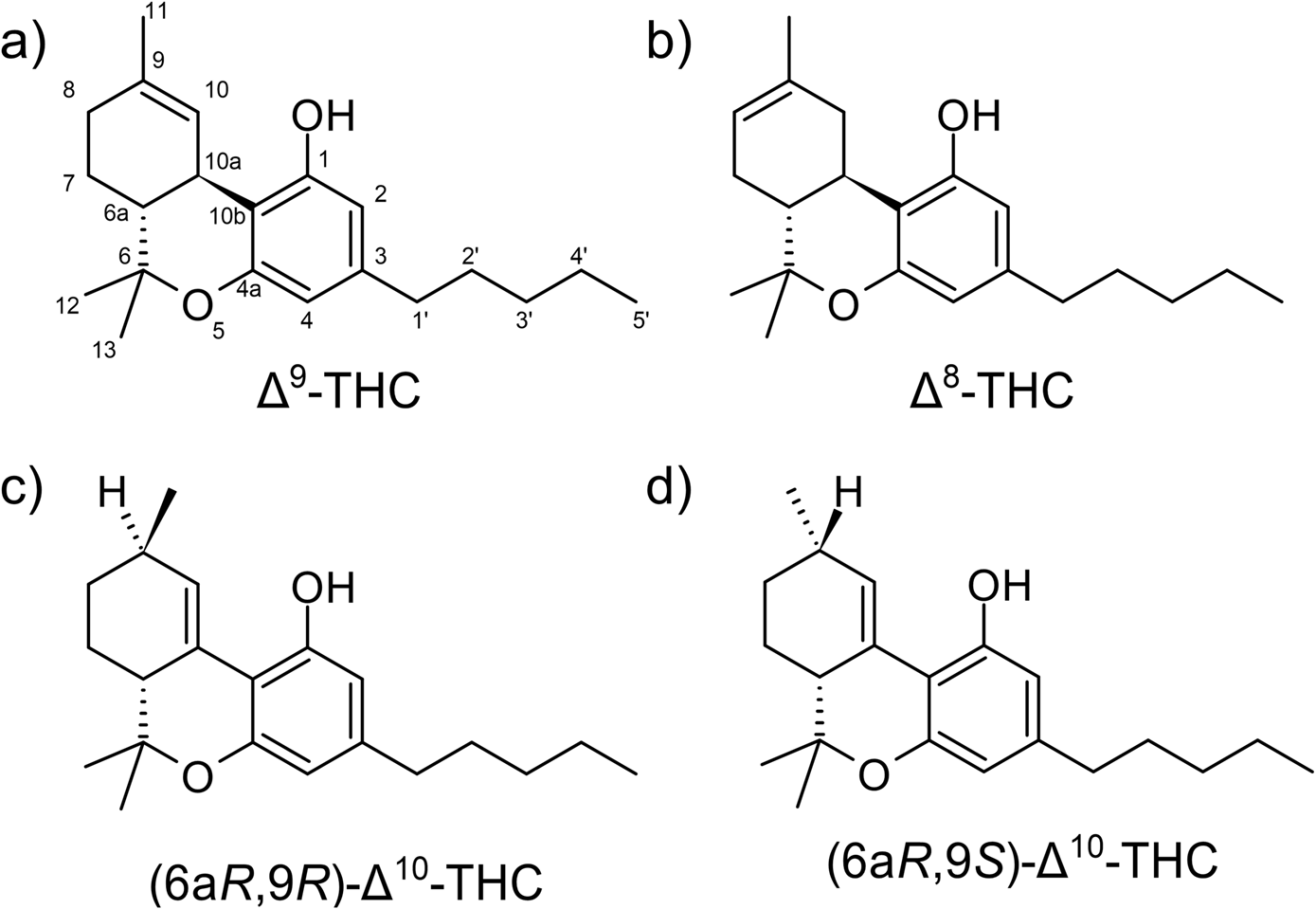
Chemical structures of (a) ∆^9^‐THC (additionally showing the molecule numbering [10]), (b) ∆^8^‐THC, and (c and d) ∆^10^‐THC diastereomers, which share the same stereoconfiguration at C6a as ∆^9^‐THC.

∆^8^‐THC was first described as a product resulting from the cyclization of CBD in the early 1940s [5, 6]. ∆^8^‐THC is also naturally present at low concentrations in cannabis and cannabis‐derived products, as was first described in 1966 [7]. In general, ∆^8^‐THC was subject to some research; however, it has been studied considerably less than ∆^9^‐THC [4]. Since the emergence of SSCs, starting off with ∆^8^‐THC around 2019 and with hexahydrocannabinol (HHC) from 2021 onwards [3, 8], SSCs are sold as legal alternatives to marijuana in various forms, ranging from e‐liquids to edibles (e.g., gummies) [8, 9]. Structurally, ∆^8^‐THC differs from ∆^9^‐THC merely in the position of the double bond in the cyclohexene ring (Figure 1). The structural similarity renders the unambiguous distinction of ∆^8^‐THC and its metabolites from ∆^9^‐THC during chemical analysis highly challenging, with serious implications in the medicolegal field [11, 12, 13, 14]. A recent nationwide study from the United States showed a widespread use of ∆^8^‐THC among adolescents, with 11.4% of 12th‐grade students reporting past‐year ∆^8^‐THC use [9].

Another SSC and isomer of Δ^9^‐THC is ∆^10^‐THC (Figure 1). With two chiral centers, four diastereomers of ∆^10^‐THC exist, of which the epimeric pair (6a*R*,9*R*)‐ and (6a*R*,9*S*)‐∆^10^‐THC (also referred to as *cis*‐ and *trans*‐∆^10^‐THC) [15] share the same stereoconfiguration at C6a as Δ^9^‐THC. ∆^10^‐THC in vaping liquids was first reported in a publication from 2021 by the US Food and Drug Administration (FDA) [16]. Outhous et al. [17] investigated products allegedly containing ∆^10^‐THC and found a great discrepancy between what was stated on the product labels and what was confirmed analytically, with ∆^10^‐THC being present in the investigated products only in trace amounts, whereas other isomers such as ∆^8^‐THC were the main constituent. In 2024, Patton et al. [18] reported that of 1300 urine samples screened positive for THC using immunoassays, 511 were positive for 11‐nor‐9‐carboxy‐∆8‐THC (Δ^8^‐THC‐COOH), most often in combination with Δ^9^‐THC, whereas Δ^10^‐THC‐COOH was detected in 77 cases. Δ^10^‐THC was, however, only detected in combination with Δ^8^‐THC or Δ^9^‐THC. Racines et al. [19] reported Δ^10^‐THC environmental exposure in hair in 24 cases with a low positivity rate of 0.4%, compared to 9.0% and 26.8% for ∆^8^‐THC and ∆^9^‐THC, respectively. Although the aforementioned studies indicate that the overall availability and use of ∆^10^‐THC is considerably lower compared to ∆^8^‐THC and ∆^9^‐THC, the same analytical challenges persist. While literature suggests similar metabolism of ∆^8^‐THC and ∆^9^‐THC [4, 18], there is currently no information on the metabolism of ∆^10^‐THC [18].

Activity‐wise, ∆^8^‐THC was shown to bind to and activate the cannabinoid receptor 1 (CB_1_) in a similar manner to ∆^9^‐THC [20, 21, 22]. Pharmacological data on ∆^10^‐THC are scarce, with one study detecting CB_1_ binding but no activation. (6a*R*,9*R*)‐ and (6a*R*,9*S*)‐∆^10^‐THC were even found to show antagonistic properties at CB_1_ [15]. Ultimately, despite the fact that the activity profiles of ∆^8^‐THC and particularly ∆^10^‐THC are not yet fully understood, these isomers are already being sold and may significantly interfere with forensic and clinical toxicological analyses [18, 22, 23]. Therefore, it is essential not only to ensure their reliable detection but also to prevent their misclassification as ∆^9^‐THC and its metabolites. Hence, this study investigated the in vitro metabolic fate of ∆^8^‐THC and ∆^10^‐THC compared to ∆^9^‐THC using human hepatocytes to assess the overlap of metabolites. For ∆^8^‐THC, additionally, forensic casework urine samples were tested to confirm the in vitro results and demonstrate analytical challenges in authentic casework samples.

## 2 Material and Methods

### 2.1 Certified Reference Material

Certified reference material of (6a*R*,10a*R*)‐∆^8^‐THC and (6a*R*,9*R*)‐∆^10^‐THC was obtained from Cayman Chemical Company (Ann Arbor, MI, USA). (6a*R*,10a*R*)‐∆^9^‐THC, (6a*R*,10a*R*)‐∆^9^‐THC‐D_3_, (±)‐11‐hydroxy‐∆^9^‐THC‐D_3_ (∆^9^‐THC‐OH‐D_3_), and (±)‐11‐nor‐9‐carboxy‐∆^9^‐THC‐D_9_ (∆^9^‐THC‐COOH‐D_9_) were from Cerilliant (Round Rock, TX, USA).

### 2.2 Human Hepatocyte Incubations

LIVERPOOL Cryosuspension human hepatocytes (mixed gender, 20‐donor pool) and thawing medium (InVitroGro HT) were obtained from BioIVT (West Sussex, UK). Williams E medium was supplemented with 2 mM of l‐glutamine and 20 mM of HEPES buffer, with the medium and supplements all purchased from Thermo Fisher Scientific (Gothenburg, Sweden). The hepatocyte incubations were conducted as described before [24], with slight adaptations. One vial containing 5 million cells was thawed and transferred into prewarmed thawing medium. After removal of the thawing medium via centrifugation, resuspension, and washing with supplemented Williams E medium, the living cells were determined using the trypan blue exclusion method, and the cells were diluted in Williams E medium to a cell concentration of 2 million cells/mL. To prevent adsorption of the highly lipophilic ∆^8^‐THC, ∆^9^‐THC, and ∆^10^‐THC to plastic labware, these drugs, as well as the positive and negative controls, were incubated in silanized HPLC glass vials (by Agilent Technologies, obtained from Scanted Nordic, Jonsered, Sweden). Incubation solutions of the drugs at 10 μM were prepared in supplemented Williams E medium, and 100 μL per vial was preheated at 37°C prior to the addition of 100 μL of the cell suspension, resulting in a 5‐μM drug concentration and 0.2 million hepatocytes per vial. The samples were incubated in duplicate at 37°C for 1 and 3 h. The reaction was quenched by adding 200 μL of ice‐cold acetonitrile (LC–MS grade) obtained from Merck (Darmstadt, Germany), containing the internal standards ∆^9^‐THC‐D_3_, ∆^9^‐THC‐OH‐D_3_, and ∆^9^‐THC‐COOH‐D_9_, and the samples were placed into the −20°C freezer for 15 min. The samples were centrifuged for 15 min at 1100 *g* and 5°C. The supernatant was transferred to HPLC vials and stored at −20°C until analysis, which was conducted within 48 h after incubation.

### 2.3 HPLC‐QToF Analysis of the Hepatocyte Samples

Samples were analyzed with high‐resolution mass spectrometry. The method used a 1290 Infinity ultra‐high‐performance liquid chromatography (UHPLC) system coupled to a 6550 iFunnel Q quadrupole time‐of‐flight mass spectrometer (QToF‐MS) by Agilent Technologies (Sundbyberg, Sweden). The HPLC system was equipped with an Acquity HSS T3 column (150 mm × 2.1 mm, ID: 1.8 μm) by Waters (Solna, Sweden). Methanol (LC–MS grade), formic acid (98%), and water (LC–MS grade) were purchased from Fisher Scientific (Gothenburg, Sweden). Mobile phases consisted of 100% water (mobile phase A) and 100% methanol (mobile phase B) with 0.05% formic acid. Chromatography was performed at 60°C, with an injection volume of 10 μL and a flow rate of 0.4 mL/min. The gradient started at 30% mobile phase A, which was held for 0.1 min. The percentage of mobile phase A was then decreased to 5% over the next 5.9 min and held for another 2 min. The system was then re‐equilibrated to the starting conditions for 1 min, resulting in a runtime of 9 min. The mass spectrometer was operated with a Dual AJS electrospray ionization (ESI) probe at a gas temperature of 300°C, with 6 L/min drying gas, 22 psi of nebulizer pressure, and 10 L/min sheath gas flow at 375°C. A capillary voltage (VCap) of 3000 V and a nozzle voltage of 1000 V were used. Data were acquired in Auto MS/MS mode with a preferred list of target molecular ions (Table S1) from which MS/MS spectra were always obtained if present.

In single cases where MS2 spectra were not obtained (due to low signal intensities), samples were run with a second method, which was better suited for glucuronides. MS2 results obtained from this method are indicated in Section 3. Full details of this second method are provided in Chapter 1 of the Supporting Information including Figure S1.

### 2.4 Data Analysis and Metabolite Annotation

Exact masses and chemical formulas of potential phase I and phase II metabolites (e.g., mono‐, di‐, tri‐hydroxylation, dehydration, glucuronidation, and combinations thereof) were calculated, and the samples were screened for these metabolites in the MassHunter Qual software (version B.07.00). A maximal mass deviation of 5 ppm in the full‐scan measurements was applied. In addition, signals were only considered if they were absent in the negative control (hepatocytes without drugs), as well as the degradation (no hepatocytes) and 0 h samples. The signals of the internal standards ∆^9^‐THC‐D_3_, ∆^9^‐THC‐OH‐D_3_, and ∆^9^‐THC‐COOH‐D_9_ were used to confirm overall instrument performance, whereas the retention times of the internal standards were compared to the corresponding metabolites for ∆^9^‐THC. The tentatively identified metabolites were further characterized by studying the MS2 spectra. For comparisons between ∆^8^‐THC, ∆^9^‐THC, and ∆^10^‐THC, the metabolite areas were summed for each analyte and timepoint, and the percentages of the total peak area of all metabolites for the individual metabolites were calculated. All calculations and graphs were prepared using Microsoft Excel (Microsoft 365, Version 2508, Stockholm, Sweden).

### 2.5 Urine Samples

Urine samples (*n* = 19) from routine cases were included in accordance with ethical approval 2018‐186/31 from the Regional Ethics Committee in Linköping, Sweden. Samples previously confirmed as containing either ∆^8^‐THC‐COOH or ∆^9^‐THC‐COOH were reanalyzed as described above for hepatocyte samples to investigate urinary metabolites of ∆^9^‐THC and ∆^8^‐THC. Urine samples were analyzed with and without hydrolysis. Hydrolyzed samples were analyzed according to a procedure described by Lindbom et al. [24]. Briefly, 50 μL of urine was fortified with 50 μL of beta‐glucuronidase (KBIO‐B‐One, Kura Biotech Finden, Puerto Varas, Chile) and hydrolyzed at room temperature for 2 h. Then 50 μL of internal standards in methanol was added before analysis. In nonhydrolyzed samples, the enzyme was exchanged for water. A 10‐μL aliquot was injected into the HPLC‐QToF system.

## 3 Results and Discussion

### 3.1 Tentative Structural Elucidation of Metabolites

Tables 1, 2, 3 show the summary of all analytical results, including the retention times, mass errors, areas at 1 and 3 h, and the major fragments. MS2 spectra for ∆^8^‐THC, ∆^9^‐THC, ∆^10^‐THC, and proposed metabolites are contained in the Supporting Information including proposed fragments (Chapters 2–5, Figures S2–S16). The retention times of the internal standards (deuterated ∆^9^‐THC, ∆^9^‐THC‐OH, and ∆^9^‐THC‐COOH) agreed with the in vitro generated metabolites for ∆^9^‐THC, thereby acting as a positive control and confirming the suitability of the applied protocol and test system. ∆^8^‐THC, ∆^9^‐THC, and ∆^10^‐THC fragmented very similarly, with predominant ions at 259.1693, 193.1223, and 135.1168 *m/z*. ∆^8^‐THC‐COOH, ∆^9^‐THC‐COOH, and ∆^10^‐THC‐COOH fragmented comparably with the detection of the fragment at 299.2006 *m/z*, which represents the THC molecule after loss of the carboxylic acid. ∆^8^‐THC‐OH and ∆^9^‐THC‐OH shared the abundant fragment at 271.1693 *m/z*, which has been described for different analytical methods for these analytes [25, 26]. Based on what is described in the literature and our results, the hydroxylation and subsequent carboxylation for these compounds are proposed at the position C11 [4]. No free ∆^10^‐THC‐OH (without glucuronidation) was detected for ∆^10^‐THC. The dihydroxylated and glucuronidated metabolite that was only detected for ∆^8^‐THC was characterized by fragments resulting from the cleavage of the glucuronide and subsequent water loss. Using the second analytical method, additionally, the fragment at 193.1223 *m/z* was detected, which indicates the locations of the hydroxylations at the ring structure and the lack of hydroxylation at the alkyl sidechain. The relatively low‐abundant metabolites ∆^8^‐THC‐OH‐GLUC and ∆^9^‐THC‐OH‐GLUC were both present as two peaks (Figure S17). As the two peaks were not chromatographically resolved and showed significant overlap for ∆^8^‐THC‐OH‐GLUC, the total area was expressed over the whole double peak (Table 1). It should be noted that this approach has limitations, as the response factors may differ between the isomers and cannot be verified in the absence of reference standards. For ∆^9^‐THC‐OH‐GLUC, separate areas are expressed in Table 2. For ∆^8^‐THC‐OH‐GLUC, the fragment at 299.2006 *m/z* (‐GLUC, ‐H_2_O, ‐CO) was detected. The absence of the ion at 193.1223 *m/z* (potentially due to the low abundance of the metabolite) does not allow for further narrowing down the location of the biotransformation. Other studies have shown before that hydroxylation predominantly happens at the position C11, as is the case for ∆^9^‐THC [4, 27]. For ∆^9^‐THC‐OH‐GLUC, no MS2 was obtained using the primary method described in Section 2.3, due to very low abundance of these metabolites. An MS2 spectrum was obtained for the first eluting peak using the second method that is described in the Supporting Information (Chapter 1). For ∆^9^‐THC‐OH‐GLUC and ∆^10^‐THC‐OH‐GLUC, the ion at 193.1223 *m/z* was found, which indicates no biotransformation at the alkyl sidechain. The presence of two metabolites for ∆^9^‐THC‐OH‐GLUC is in accordance with a study by Hassenberg et al. [28], who confirmed the presence of a phenolic and an alcoholic glucuronide in vitro and in vivo. ∆^10^‐THC‐OH‐GLUC was not only significantly higher in abundance compared to the ∆^8^‐ and ∆^9^‐THC samples but also present as a single peak, indicating that only one of the glucuronides is present for this isomer. The most abundant fragments for ∆^8^‐THC‐COOH‐GLUC and ∆^9^‐THC‐COOH‐GLUC resulted from the cleavage of the glucuronide (345.2060 *m/z*) and subsequent water loss (327.1955 *m/z*). Additionally, the ion at *m/z* 299.2006 was detected, as also observed for ∆^8^‐THC‐COOH and ∆^9^‐THC‐COOH.

**TABLE 1:** Analytical results for ∆^8^‐THC. “RT”: retention time, “Theor. Mass”: theoretical mass, “Max. mass error”: maximal mass error. Areas are reported as averages from *n* = 2 samples.

Analyte/tentative metabolite	Chemical formula	RT (min)	Theor. mass [M + H]^+^ (*m/z*)	Max. mass error (ppm)	Area at 1 h	Area at 3 h	Major fragments (*m/z*)
∆^8^‐THC	C_21_H_30_O_2_	6.15	315.2318	1.86	1.79E+06	6.98E+05	259, 193, 135, 93
∆^8^‐THC‐OH	C_21_H_30_O_3_	4.32	331.2268	1.42	1.27E+05	3.93E+04	313, 271, 257, 201
∆^8^‐THC‐OH‐GLUC	C_27_H_38_O_9_	3.02	507.2589	−1.81	9.84E+04	1.06E+05	331, 313, 299, 257
∆^8^‐THC‐COOH	C_21_H_28_O_4_	4.35	345.2060	0.94	2.18E+05	2.06E+05	327, 299, 257, 193
∆^8^‐THC‐COOH‐GLUC	C_27_H_36_O_10_	2.65	521.2381	2.5	5.69E+04	3.07E+05	345, 327, 257, 193
∆^8^‐THC‐DiOH‐GLUC	C_27_H_38_O_10_	2.99	523.2538	−0.76	8.83E+04	1.26E+05	347, 329, 193^a^

^a^ Fragment detected using the method described in Chapter 1 of the Supporting Information.

**TABLE 2:** Analytical results for ∆^9^‐THC. “RT”: retention time, “Theor. Mass”: theoretical mass, “Max. mass error”: maximal mass error. Areas are reported as averages from *n* = 2 samples.

Analyte/tentative metabolite	Chemical formula	RT (min)	Theor. mass [M + H]^+^ (*m/z*)	Max. mass error (ppm)	Area at 1 h	Area at 3 h	Major fragments (*m/z*)
∆^9^‐THC	C_21_H_30_O_2_	6.01	315.2318	0.77	2.13E+06	1.19E+06	259, 193, 135, 93
∆^9^‐THC‐OH	C_21_H_30_O_3_	4.28	331.2268	3.6	2.19E+05	9.17E+04	313, 271, 201, 193, 175
∆^9^‐THC‐OH‐GLUC 1	C_27_H_38_O_9_	2.80	507.2589	2.75	4.34E+04	9.49E+04	353^a^, 341^a^, 193^a^
∆^9^‐THC‐OH‐GLUC 2	C_27_H_38_O_9_	3.09	507.2589	−4.8	4.58E+04	5.05E+04	—
∆^9^‐THC‐COOH	C_21_H_28_O_4_	4.50	345.2060	−4.76	1.55E+05	4.43E+04	327, 299, 257, 193
∆^9^‐THC‐COOH‐GLUC	C_27_H_36_O_10_	2.88	521.2381	−3.06	1.39E+05	2.00E+05	345, 327, 299, 283, 257

^a^ Fragment detected using the method described in Chapter 1 of the Supporting Information.

**TABLE 3:** Analytical results for ∆^10^‐THC. “RT”: retention time, “Theor. Mass”: theoretical mass, “Max. mass error”: maximal mass error. Areas are reported as averages from *n* = 2 samples.

Analyte/tentative metabolite	Chemical formula	RT (min)	Theor. mass [M + H]^+^ (*m/z*)	Max. mass error (ppm)	Area at 1 h	Area at 3 h	Major fragments (*m/z*)
∆^10^‐THC	C_21_H_30_O_2_	6.38	315.2318	2.1	4.49E+06	1.65E+06	259, 193, 135, 93
∆^10^‐THC‐GLUC	C_27_H_38_O_8_	3.90	491.2639	−1.82	4.79E+05	3.73E+05	315, 233, 193, 135
∆^10^‐THC‐OH‐GLUC	C_27_H_38_O_9_	3.13	507.2589	−1.64	4.64E+05	5.78E+05	331, 257, 193, 177
∆^10^‐THC‐COOH	C_21_H_28_O_4_	4.60	345.2060	−4.97	7.17E+04	8.55E+04	327, 299, 231

### 3.2 Relative Metabolite Abundances

**FIGURE 2:**
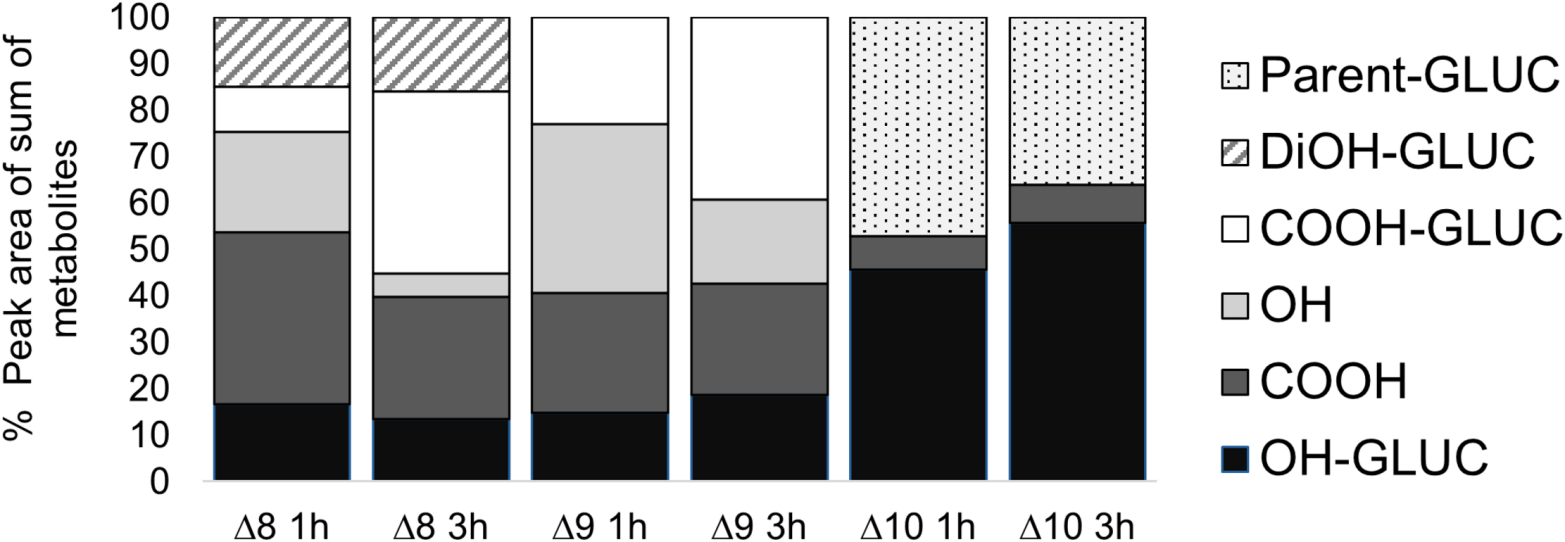
Diagram showing the different percentages of the observed metabolites compared to the sum of the area of all metabolites per isomer and timepoint (average of *n* = 2 measurements). ∆^8^‐THC‐OH‐GLUC and ∆^9^‐THC‐OH‐GLUC were present as two partially (∆^8^) and fully (∆^9^) resolved peaks; for simplicity, they are expressed as the sum of the two peaks.

Figure 2 shows the corresponding metabolite distributions (expressed as percentages of the normalized areas; sum of all metabolites equals 100%) for ∆^8^‐THC, ∆^9^‐THC, and ∆^10^‐THC. ∆^9^‐THC and ∆^8^‐THC are metabolized in a similar manner, as they were predominantly hydroxylated and further carboxylated, resulting in the formation of ∆^8/9^‐THC‐OH and ∆^8/9^‐THC‐COOH. The secondary metabolites ∆^8^‐THC‐COOH‐GLUC and ∆^9^‐THC‐COOH‐GLUC were the major metabolites at the 3‐h timepoints. These results are in line with urine metabolites detected in casework samples [11, 13, 23] and literature [4]. In the presented study, for ∆^8^‐THC, additionally, a dihydroxylated and glucuronidated metabolite was detected, which was not observed for ∆^9^‐THC. Overall, for ∆^9^‐THC, over 80 metabolites have been described [27], for example, (8*R*,11)‐dihydroxy‐∆^9^‐THC, as has been confirmed in authentic plasma samples [29]. Other studies reporting more metabolites for ∆^8^‐THC and ∆^9^‐THC used pooled human liver microsomes [30, 31]. Differences might result from the different in vitro systems used as well as analytical method sensitivity.

### 3.3 ∆^10^‐THC Metabolism Compared With ∆^8^‐THC and ∆^9^‐THC

When comparing ∆^10^‐THC to ∆^8^‐THC and ∆^9^‐THC, it was found that whereas ∆^8^‐THC and ∆^9^‐THC metabolized comparably, the metabolism of ∆^10^‐THC differed considerably. The major metabolites observed for ∆^10^‐THC were ∆^10^‐THC‐OH‐GLUC and the glucuronidated parent compound ∆^10^‐THC‐GLUC. The latter was not detected in the hepatocyte samples for either ∆^8^‐THC or ∆^9^‐THC. A low‐abundant metabolite corresponding to ∆^10^‐THC‐COOH was found; however, it strongly differed in its abundance compared to ∆^8^‐THC and ∆^9^‐THC. Consequently, it was observed that the monohydroxylated metabolite appears not to be further oxidized to the carboxylic acid, which is in strong contrast to the metabolic pathways observed for ∆^8^‐THC and ∆^9^‐THC. Further distinguishing the metabolism of ∆^10^‐THC was the observation of only minor differences between the two timepoints regarding the detected metabolites and their abundances (Figure 2). In addition, only one peak was observed for ∆^10^‐THC‐OH‐GLUC.

**FIGURE 3:**
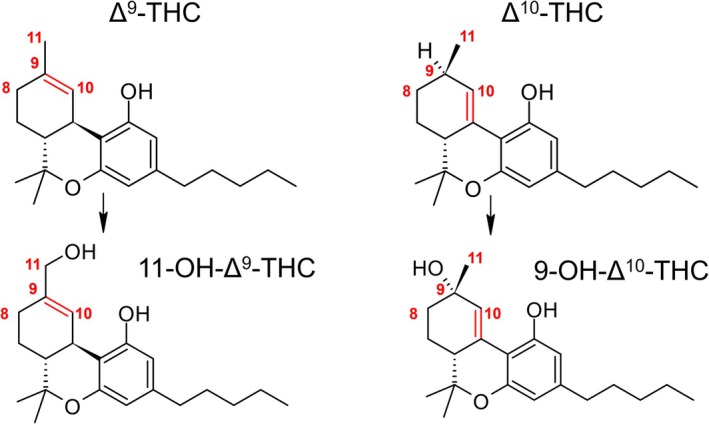
Metabolic pathway of ∆^9^‐THC to ∆^9^‐THC‐OH compared to the proposed metabolic pathway for the herein investigated (6a*R*,9*R*)‐∆^10^‐THC.

A possible explanation for the observed differences could be that for ∆^10^‐THC, the hydroxylation takes place at another site than the terminal C11 position, as is the predominant metabolic site for ∆^8^‐THC and ∆^9^‐THC [4, 27, 31]. The allylic C11 position is metabolically favored for ∆^9^‐THC and ∆^8^‐THC. Hydroxylation has also been shown before at the allylic position C8 [27, 31]. The preference for the allylic sites for metabolism has also been described for other cannabinoids such as CBD [32]. For ∆^10^‐THC, due to the different site of the double bond, allylic sites are shifted to the C9 and C6a positions. Unfortunately, there was no MS2 spectrum measured for the ∆^10^‐THC‐OH metabolite due to the very low abundance, and the MS2 spectrum of ∆^10^‐THC‐OH‐GLUC did not allow for further structure elucidation. Based on the overall results and steric considerations, we hypothesized that hydroxylation for ∆^10^‐THC takes place at the position C9, as visualized in Figure 3. Further supporting the hypothesis of hydroxylation predominantly taking place at a nonterminal position for ∆^10^‐THC, and hence at a different site than C11, is that (practically) no carboxylic acid was detected, as further oxidation of the hydroxy group at the C9 position (or the other allylic site C6a) would require breakage of C–C bonds. The traces of a supposed carboxylic acid metabolite for ∆^10^‐THC imply that some hydroxylation and subsequent oxidation to the carboxylic acid may happen at the C11 position (or potentially the other terminal site C5′) but to a considerably lower degree compared to hydroxylation at the hypothesized other site. Furthermore, the ∆^10^‐THC‐OH‐GLUC metabolite for ∆^10^‐THC was present as a single peak, in contrast to the two peaks observed for ∆^8^‐THC and ∆^9^‐THC. This may indicate that only the phenolic glucuronide is formed, whereas the hydroxy group at the presumed C9 position is not glucuronidated. The lack of further glucuronidation at this site might be explained by greater steric hindrance compared to hydroxylation at the terminal C11 position. To confirm the herein proposed sites of metabolism for ∆^10^‐THC, the synthesis and analysis of corresponding reference standards would be required.

Data on the metabolism of ∆^10^‐THC are very limited. One investigation (Cayman Novel Psychoactive Substance Metabolism Monograph) [33] found three monohydroxylated metabolites for ∆^10^‐THC using HLM, with suggested hydroxylations at the ring system but also the pentyl sidechain. Patton et al. [18] confirmed the presence of Δ^10^‐THC‐COOH in 20 authentic urine samples. For ∆^10^‐THC, four different diastereomers exist, of which (6a*R*,9*R*)‐∆^10^‐THC was investigated herein. This is one of the epimers that share the same stereochemistry at C6a as ∆^9^‐THC. Future research should also investigate and compare the metabolism of the other epimer (6a*R*,9*S*)‐∆^10^‐THC, as metabolism may differ.

Overall, this study confirmed the similar metabolism for ∆^9^‐THC and ∆^8^‐THC. Further, our data indicate that suitable screening targets for ∆^10^‐THC are the unchanged parent after hydrolysis as well as the monohydroxylated metabolite. These considerable differences in metabolism are of great importance, considering that misclassification of ∆^10^‐THC and its metabolites as ∆^9^‐THC could lead to drawing false conclusions in clinical and forensic analyses. This is further exacerbated considering that ∆^10^‐THC might show different activity profiles compared to ∆^9^‐THC.

### 3.4 Urine Samples

Nineteen urine samples containing metabolites from only ∆^8^‐THC, only ∆^9^‐THC, or a mixture were analyzed with the same method as the hepatocyte incubations. The major metabolites found were THC‐COOH, together with 11‐OH‐THC, and THC itself mainly as glucuronides in the unhydrolyzed samples. This is largely in accordance with the hepatocyte results. The dihydroxylated metabolites were only identified in three of the hydrolyzed urine samples. In the human hepatocyte samples, the dihydroxylated and glucuronidated metabolite was only found for ∆^8^‐THC. Chromatograms from a sample containing both ∆^8^‐THC and ∆^9^‐THC OH‐ and COOH‐ metabolites are shown in Figure S18. The method was capable of separating the isomers of the hydroxylated and carboxylated metabolites. Unfortunately, no authentic urine sample containing ∆^10^‐THC metabolites was available at the time of the study.

### 3.5 Limitations

Glucuronides are not often subjected to routine analyses and research, as, for instance, urine samples are most often hydrolyzed prior to analysis. Accordingly, the presented method, which is used for routine analysis of ∆^9^‐THC, is not optimized for glucuronides. To further investigate the specific structure of the herein observed glucuronides, reference standards would be required. Furthermore, the analytical method desirably should be further optimized for glucuronides (e.g., source parameters and collision energies) to obtain more informative MS2 spectra. This study used human hepatocytes, a recognized in vitro model, to assess the metabolism of Δ^10^‐THC [34]. The presented results should be confirmed by measuring reference standards of all the proposed metabolites. Further, the metabolic profile should be confirmed in vivo by measuring human urine samples after Δ^10^‐THC intake.

## 4 Conclusion

This study investigated the in vitro metabolism of the recently detected ∆^10^‐THC, compared to its isomers ∆^8^‐THC and ∆^9^‐THC. Significant differences in metabolism were found, with high relevance in clinical and forensic toxicological investigations. Although the structural closeness comprises a significant challenge, the different metabolic fates observed for ∆^10^‐THC compared to ∆^8^‐THC and ∆^9^‐THC bear additional room for misclassification and misinterpretation of analytical results.

## Author Contributions

**Robert Kronstrand:** conceptualization, methodology, visualization, formal analysis, writing – review and editing. **Henrik Green:** conceptualization, methodology, supervision, writing – review and editing. **Markus Loh:** formal analysis, investigation, writing – review and editing. **Fabian Rüttimann:** formal analysis, investigation, writing – review and editing. **Manuela Carla Monti:** conceptualization, methodology, visualization, formal analysis, investigation, supervision, writing – original draft, writing – review and editing.

## Funding

This study was supported by the Schweizerischer Nationalfonds zur Förderung der Wissenschaftlichen Forschung within the Postdoc.Mobility (Grant Number 217677 to MCM) and Return CH Postdoc.Mobility (Grant Number 235057 to MCM) funding schemes.

## Conflicts of Interest

The authors declare no conflicts of interest.

## Supporting information


**Table S1:** Preferred Ion list.
**Figure S1:** Further details on the second analytical method.
**Figure S2:** ∆^8^‐THC.
**Figure S3:** ∆^9^‐THC.
**Figure S4:** ∆^10^‐THC.
**Figure S5:** ∆^8^‐THC‐OH.
**Figure S6:** ∆^8^‐THC‐OH‐GLUC: (a) zoomed in version also showing the ion at 299.1995 *m/z*, (b) whole mass range.
**Figure S7:** ∆^8^‐THC‐COOH.
**Figure S8:** ∆^8^‐THC‐COOH‐GLUC.
**Figure S9:** ∆^8^‐THC‐DiOH‐GLUC.
**Figure S10:** ∆^9^‐THC‐OH.
**Figure S11:** ∆^9^‐THC‐OH‐GLUC 1.
**Figure S12:** ∆^9^‐THC‐COOH.
**Figure S13:** ∆^9^‐THC‐COOH‐GLUC.
**Figure S14:** ∆^10^‐THC‐GLUC.
**Figure S15:** ∆^10^‐THC‐OH‐GLUC.
**Figure S16:** ∆^10^‐THC‐COOH.
**Figure S17:** Chromatograms showing the OH‐GLUC metabolites for ∆^8^‐THC, ∆^9^‐THC, and ∆^10^‐THC. *Please note that the chromatograms are auto‐scaled*.
**Figure S18:** Chromatograms of an authentic urine sample.

## Data Availability

The data that support the findings of this study are available from the corresponding author upon reasonable request.
