# Supplementary material for: In Vitro Metabolism of Δ8‐, Δ9‐, and Δ10‐THC in Human Hepatocytes: Distinct Δ10‐THC Biotransformation and Implications for Drug Testing
Source: Drug Test Anal. 2026 Jun 2;18(8):1114–21. doi: 10.1002/dta.70101 (PMC13432780; doi:10.1002/dta.70101)
Supplement: Supplementary file 1 — Table S1: Preferred Ion list. Figure S1: Further details on the second analytical method. Figure S2: ∆8‐THC. Figure S3: ∆9‐THC. Figure S4: ∆10‐THC. Figure S5: ∆8‐THC‐OH. Figure S6: ∆8‐THC‐OH‐GLUC: (a) zoomed in version also showing the ion at 299.1995 m/z, (b) whole mass range. Figure S7: ∆8‐THC‐COOH. Figure S8: ∆8‐THC‐COOH‐GLUC. Figure S9: ∆8‐THC‐DiOH‐GLUC. Figure S10: ∆9‐THC‐OH. Figure S11: ∆9‐THC‐OH‐GLUC 1. Figure S12: ∆9‐THC‐COOH. Figure S13: ∆9‐THC‐COOH‐GLUC. Figure S14: ∆10‐THC‐GLUC. Figure S15: ∆10‐THC‐OH‐GLUC. Figure S16: ∆10‐THC‐COOH. Figure S17: Chromatograms showing the OH‐GLUC metabolites for ∆8‐THC, ∆9‐THC, and ∆10‐THC. Please note that the chromatograms are auto‐scaled. Figure S18: Chromatograms of an authentic urine sample. [file DTA-18-1114-s001.docx]

Supplementary Material:

***In vitro* metabolism of Δ^8^-, Δ^9^-, and Δ^10^-THC in human hepatocytes: distinct Δ^10^-THC biotransformation and implications for drug testing**

Robert Kronstrand^a,b^, Henrik Green^a,b^, Markus Loh^a,c^, Fabian Rüttimann^a,c^, Manuela Carla Monti^a,d^

^a^Department of Biomedical and Clinical Science, Division of Clinical Chemistry and Pharmacology, Linköping University, Linköping, Sweden

^b^Department of Forensic Genetics and Forensic Toxicology, National Board of Forensic Medicine, Linköping, Sweden

^c^School of Life Sciences FHNW, University of Applied Sciences and Arts Northwestern Switzerland, Muttenz, Switzerland

^d^Institute of Forensic Medicine, Department of Biomedical Engineering, University of Basel, Switzerland

*Corresponding author: [manuela.monti@unibas.ch](mailto:manuela.monti@unibas.ch)

**Chapter 1:** Mass spectrometer instrument settings p. 2

**Chapter 2:** MS2 spectra of ∆^8^-THC, ∆^9^-THC, and ∆^10^-THC p. 3

**Chapter 3:** MS2 spectra of metabolites of ∆^8^-THC p. 4-6

**Chapter 4:** MS2 spectra of metabolites of ∆^9^-THC p. 7-8

**Chapter 5:** MS2 spectra of metabolites of ∆^10^-THC p. 9

**Chapter 6:** OH-GLUC metabolites of ∆^8^-THC, ∆^9^-THC, and ∆^10^-THC p. 10

**Chapter 7:** Example chromatograms of authentic urine sample p. 11

Chapter 1: Mass spectrometer instrument settings

**Table S1:** Preferred Ion list.

| ***m/z***  **[M+H]^+^** | **Delta *m/z* (ppm)** | **Ret. Time (min)** | **Delta Ret. Time (min)** | **Collision Energy** | **Anticipated analyte** | **Formula** |
| --- | --- | --- | --- | --- | --- | --- |
| 315.2319 | 20 | 6.21 | 0.5 | 20 | THC | C_21_H_30_O_2_ |
| 345.206 | 20 | 4.69 | 0.5 | 20 | THC-COOH | C_21_H_28_O_4_ |
| 331.2268 | 20 | 4.46 | 0.5 | 20 | 11-OH-THC | C_21_H_30_O_3_ |
| 521.2381 | 20 | 2.83 | 0.5 | 20 | THCCOOH gluc | C_27_H_36_O_10_ |
| 521.2381 | 20 | 3.08 | 0.5 | 20 | THCCOOH gluc | C_27_H_36_O_10_ |
| 507.2589 | 20 | 2.76 | 0.5 | 20 | OH-THC gluc | C_27_H_38_O_9_ |
| 491.2639 | 20 | 4.05 | 0.5 | 20 | THC gluc | C_27_H_38_O_8_ |
| 523.2538 | 20 | 3.21 | 0.5 | 20 | DiOH THC gluc | C_27_H_38_O_10_ |
| 347.2217 | 20 | 3.79 | 0.5 | 20 | DiOH | C_21_H_30_O_4_ |
| 347.2217 | 20 | 4.78 | 0.5 | 20 | DiOH | C_21_H_30_O_4_ |

**Analytical method used for additional MS2 spectra**

The hepatocyte samples were analyzed employing a complementary method to the one described in the main article. A 1290 Infinity ultra high-performance liquid chromatography (UHPLC) system coupled to a 6550 iFunnel Q quadrupole time-of-flight mass spectrometer (QToF-MS) by Agilent technologies (Sundbyberg, Sweden) was used. The HPLC system was equipped with the same column as described in the main article, namely, a Acquity HSS T3 column (150mm x 2.1mm, ID: 1.8µm) by Waters (Solna, Sweden).

Mobile phases consisted of 100% water with 0.5% formic acid (mobile phase A) and 100% methanol with 0.5% formic acid (mobile phase B). Chromatography was performed at 60 °C. An injection volume of 5 µL was used.

In **Fig. S1** the gradient, mass acquisition, and source parameters are presented.

**Fig. S1** Further details on the second analytical method.


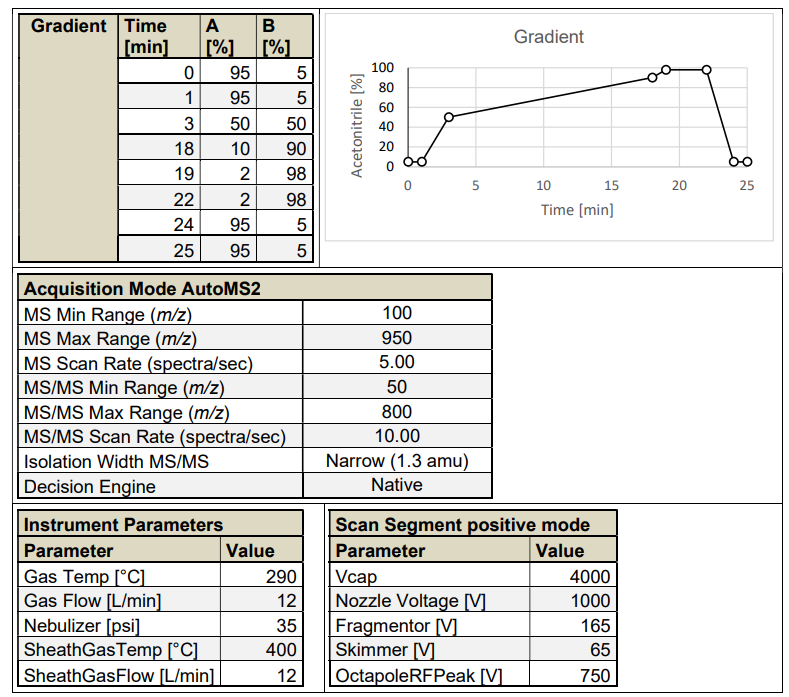


**Chapter 2:** MS2 spectra of ∆^8^-THC, ∆^9^-THC, and ∆^10^-THC

**Fig. S2:** ∆^8^-THC


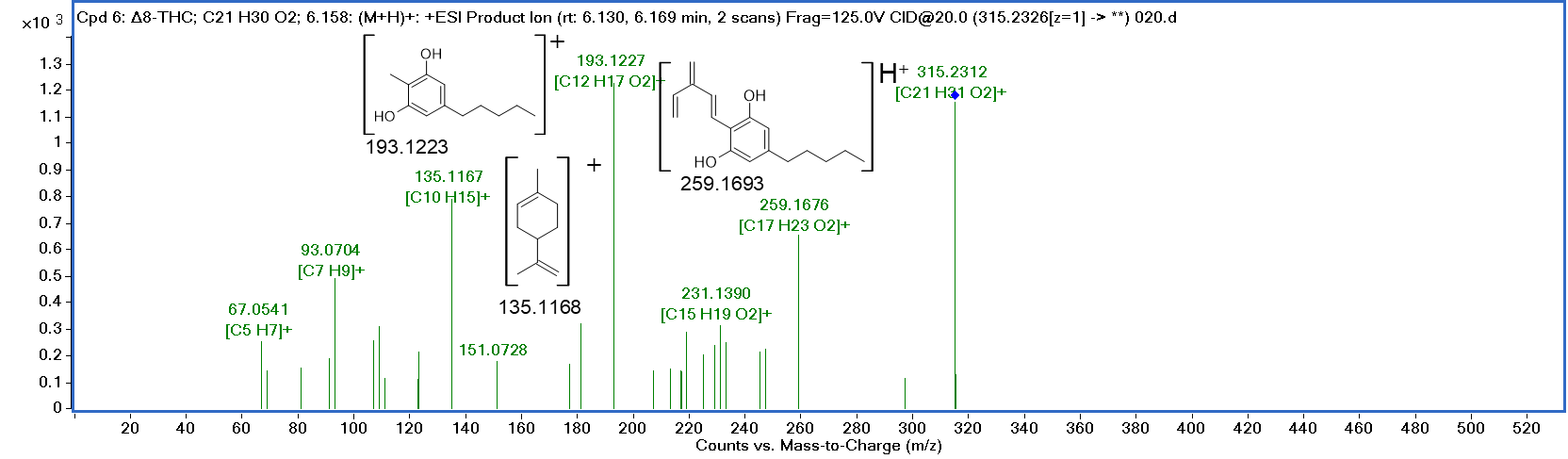


**Fig. S3:** ∆^9^-THC

**
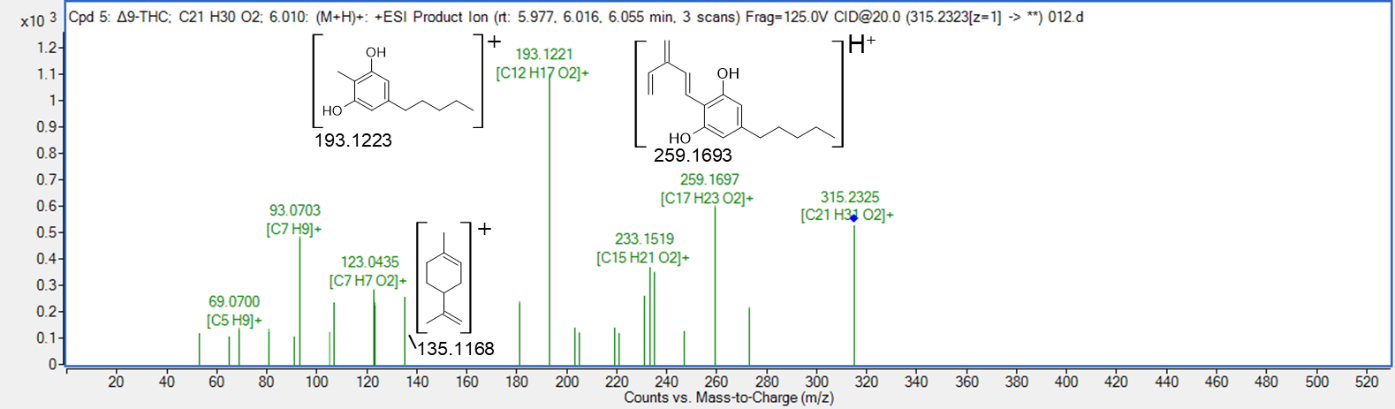
**

**Fig. S4:** ∆^10^-THC


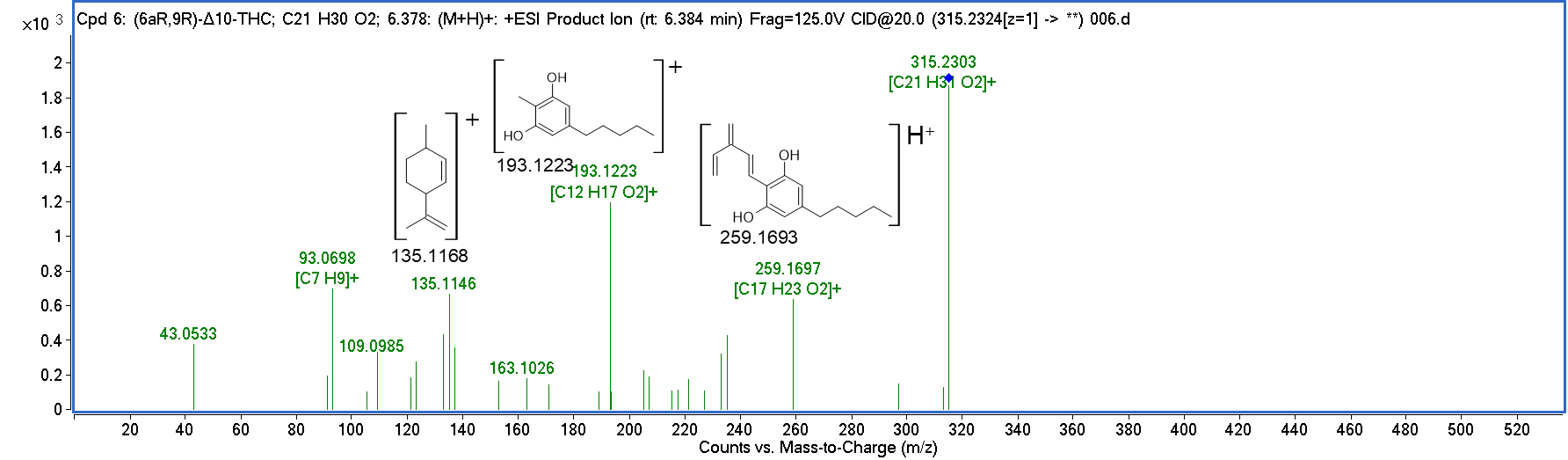


**Chapter 3:** MS2 spectra of metabolites of ∆^8^-THC

**Fig. S5:** ∆^8^-THC-OH


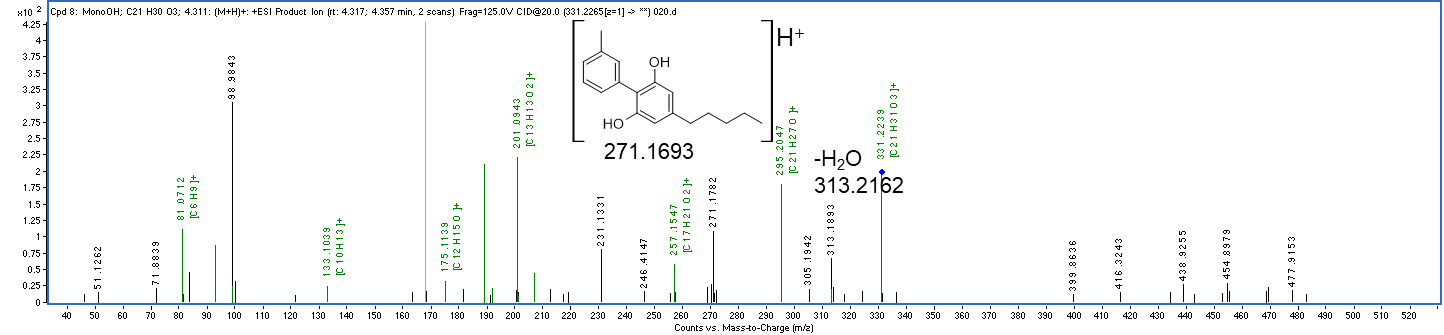


**Fig. S6:** ∆^8^-THC-OH-GLUC: a) zoomed in version also showing the ion at 299.1995 *m/z*, b) whole mass range

a)


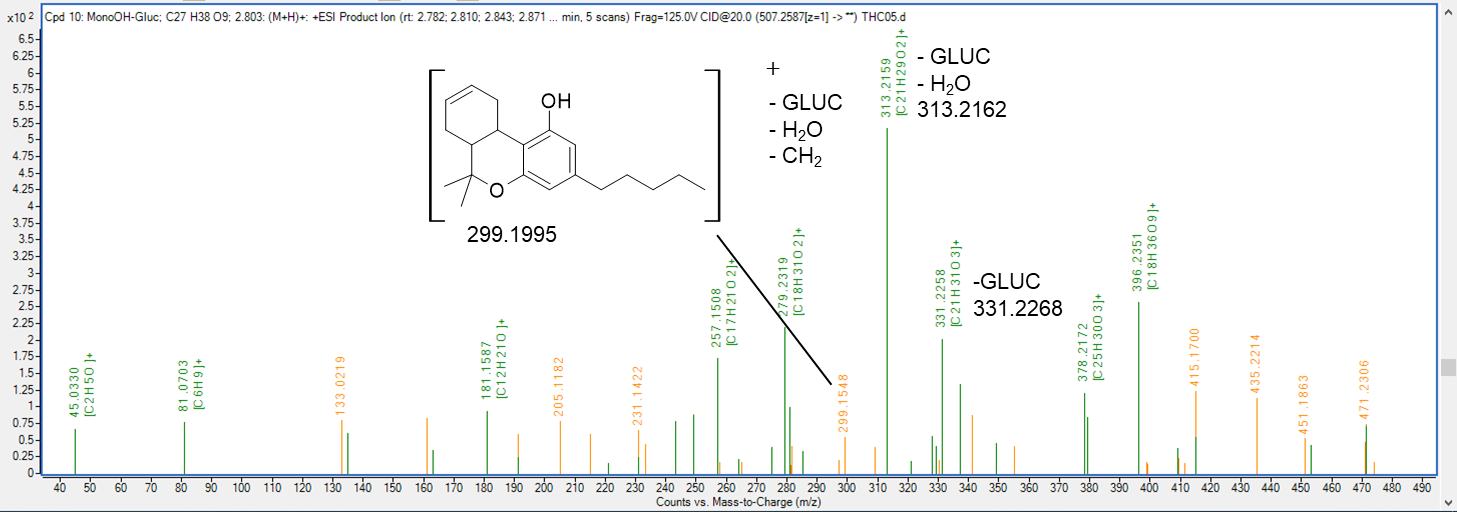


b)


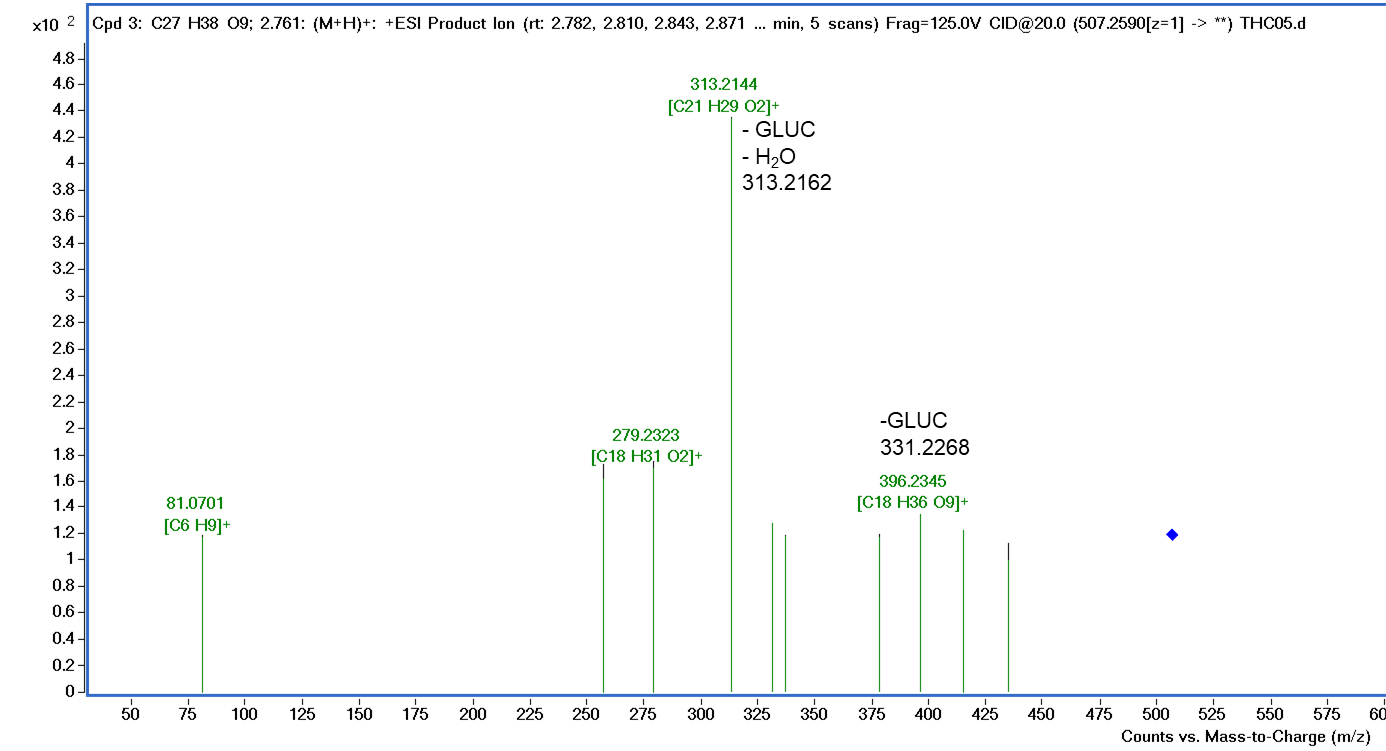


**Fig. S7:** ∆^8^-THC-COOH


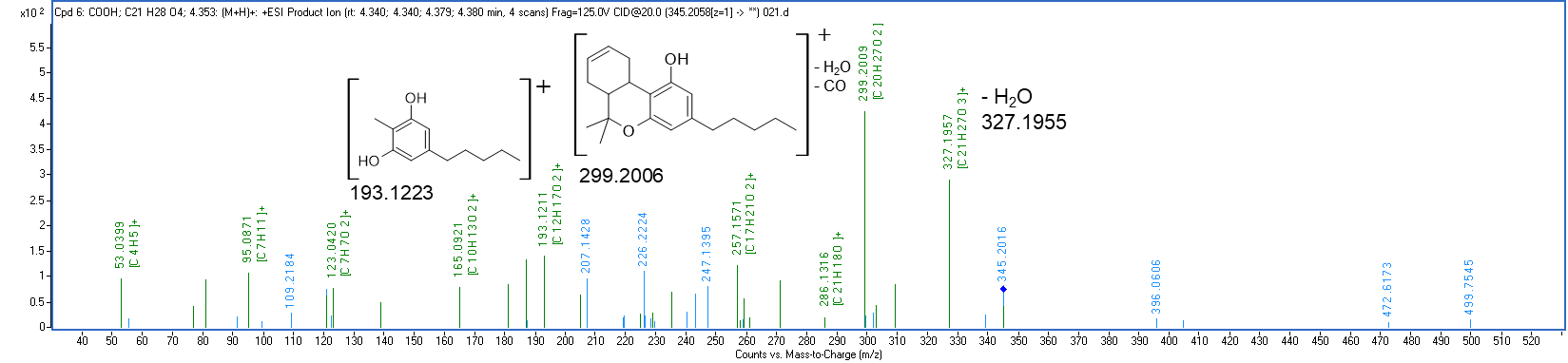


**Fig. S8:** ∆^8^-THC-COOH-GLUC


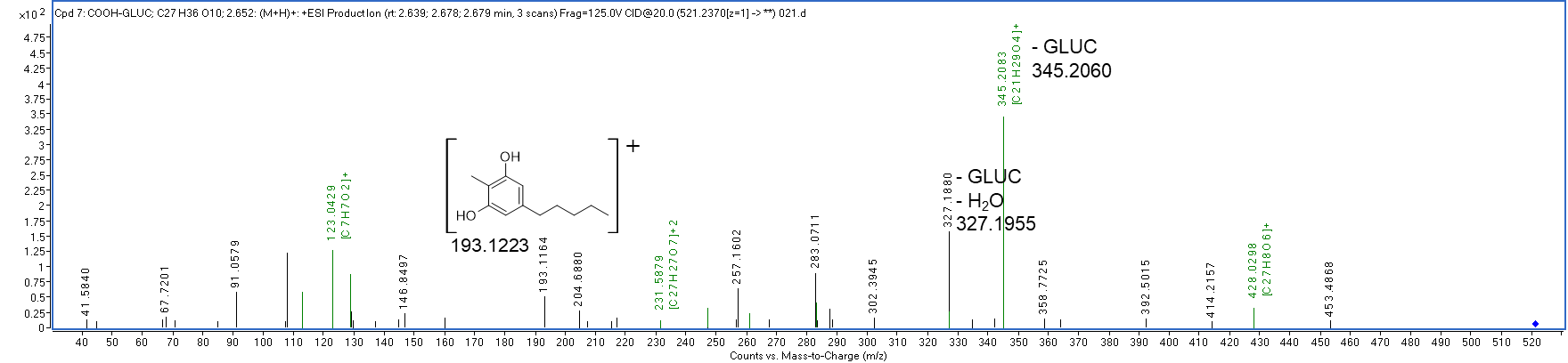


**Fig. S9:** ∆^8^-THC-DiOH-GLUC


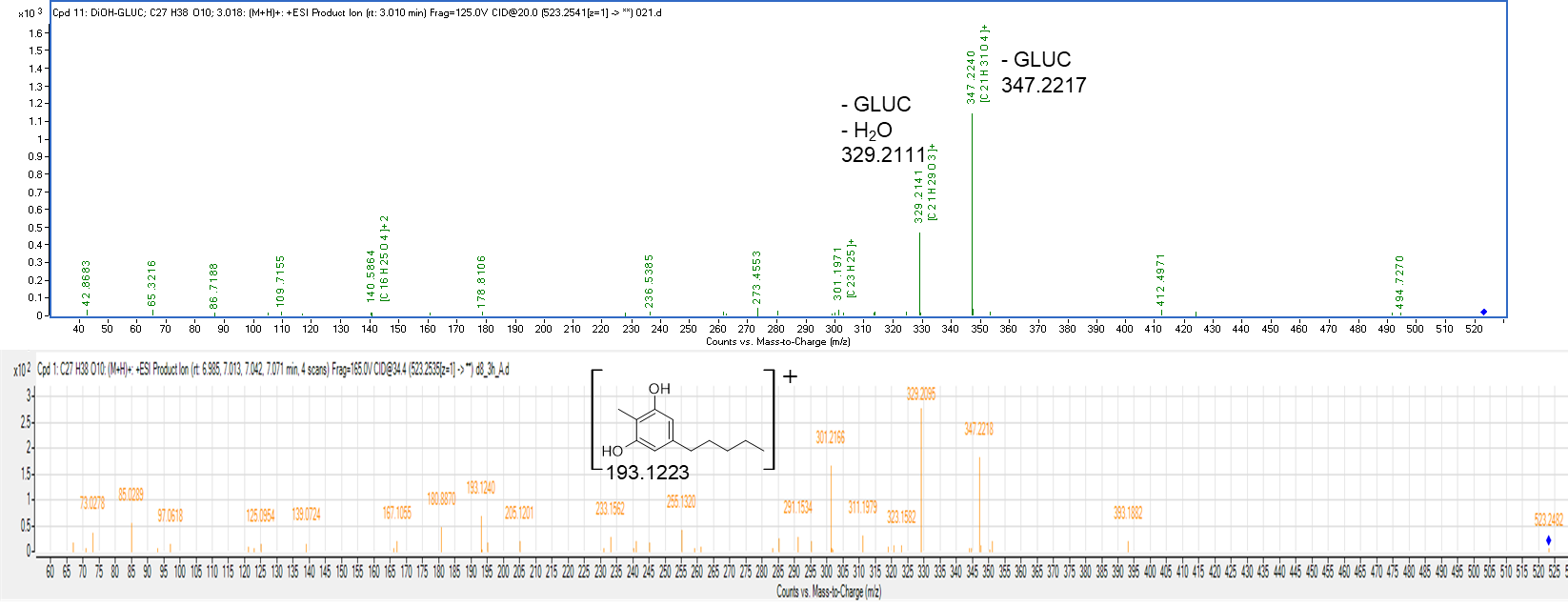


*Note: The lower spectrum was obtained using the separate method described in supplementary material chapter 1.*

**Chapter 4:** MS2 spectra of metabolites of ∆^9^-THC

**Fig. S10:** ∆^9^-THC-OH


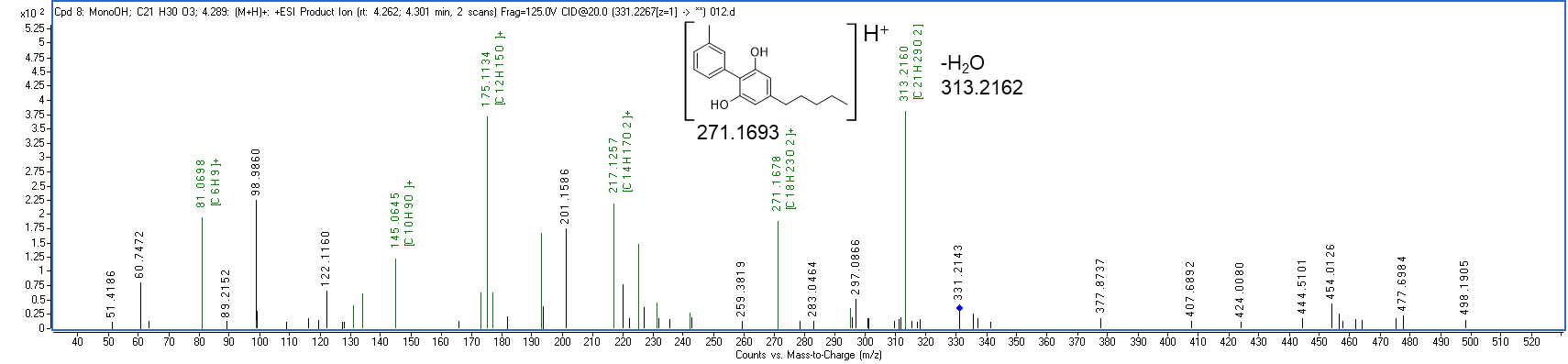


**Fig. S11:** ∆^9^-THC-OH-GLUC 1


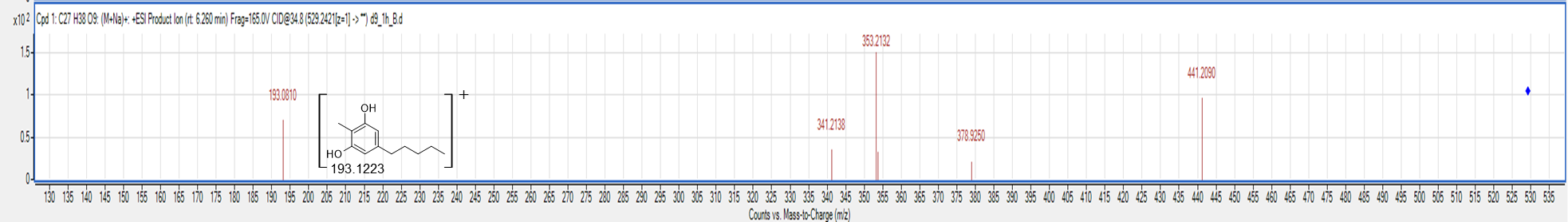


*Note: Spectrum was obtained using the separate method described in supplementary material chapter 1.*

**Fig. S12:** ∆^9^-THC-COOH


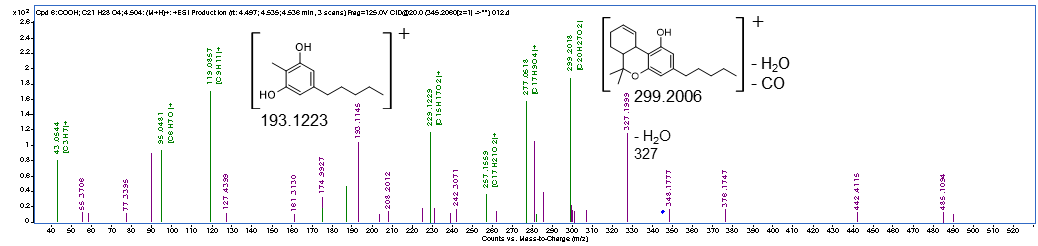


**Fig. S13:** ∆^9^-THC-COOH-GLUC


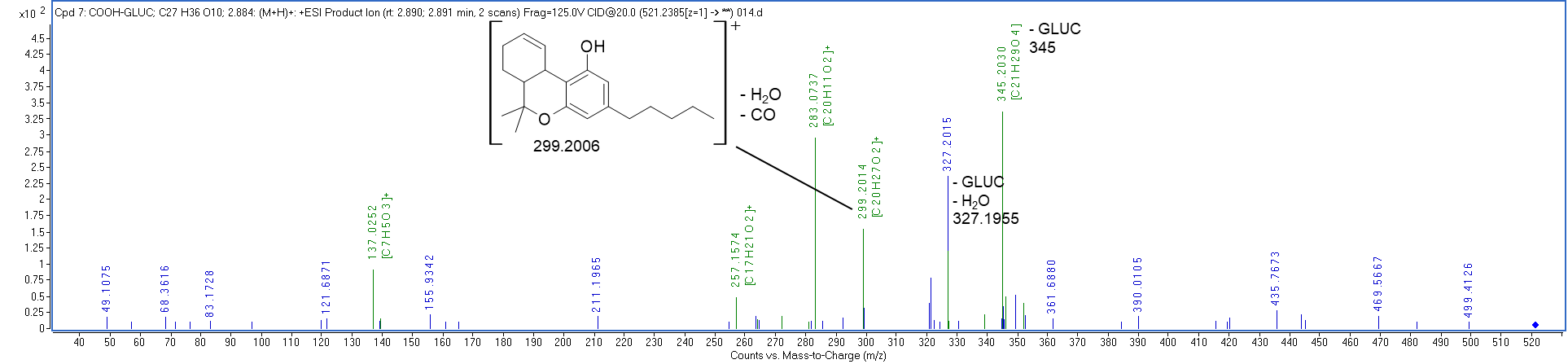


**Chapter 5:** MS2 spectra of metabolites of ∆^10^-THC

**Fig. S14:** ∆^10^-THC-GLUC


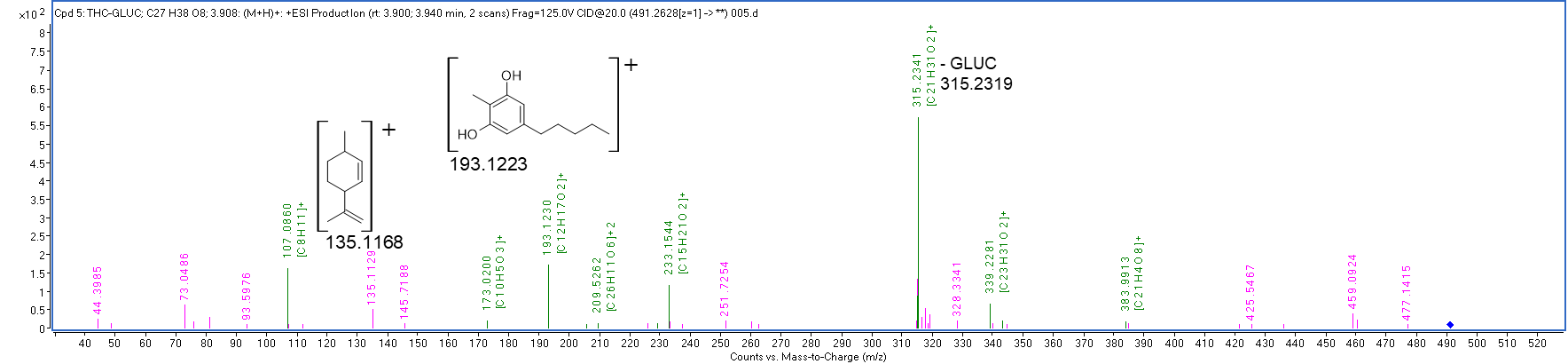


**Fig. S15:** ∆^10^-THC-OH-GLUC

**
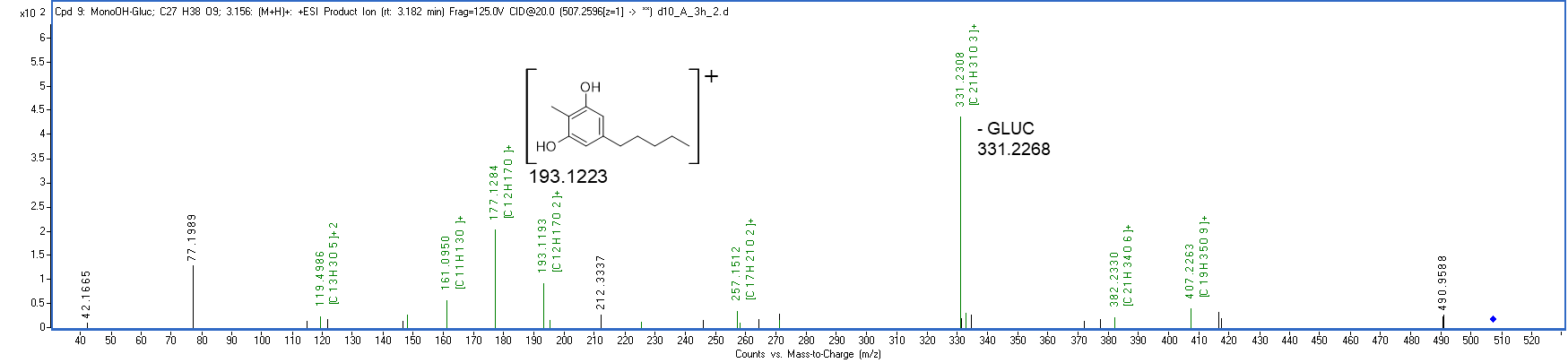
**

**Fig. S16:** ∆^10^-THC-COOH


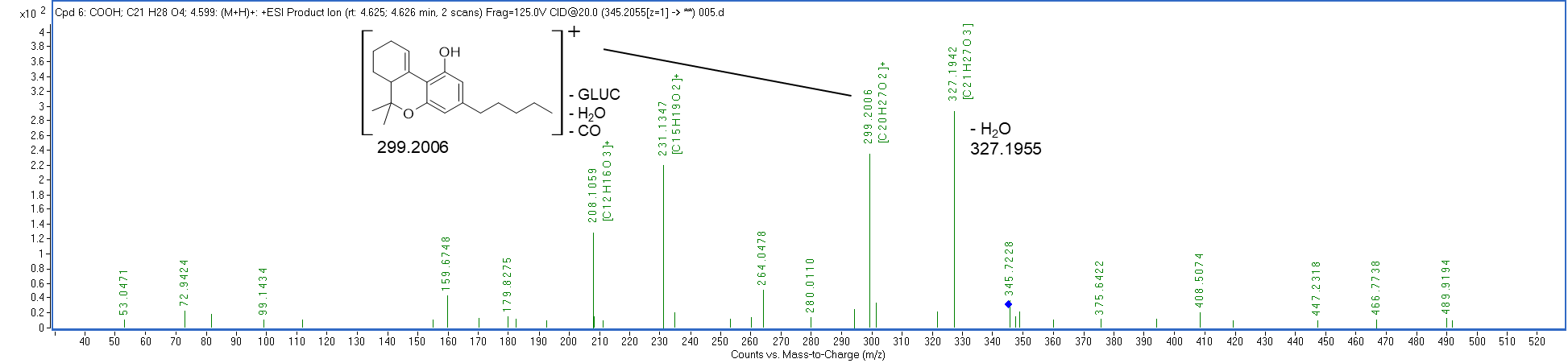


**Chapter 6:** OH-GLUC metabolites of ∆^8^-THC, ∆^9^-THC, and ∆^10^-THC

**Fig. S17:** Chromatograms showing the OH-GLUC metabolites for ∆^8^-THC, ∆^9^-THC, and ∆^10^-THC. *Please note that the chromatograms are auto-scaled.*


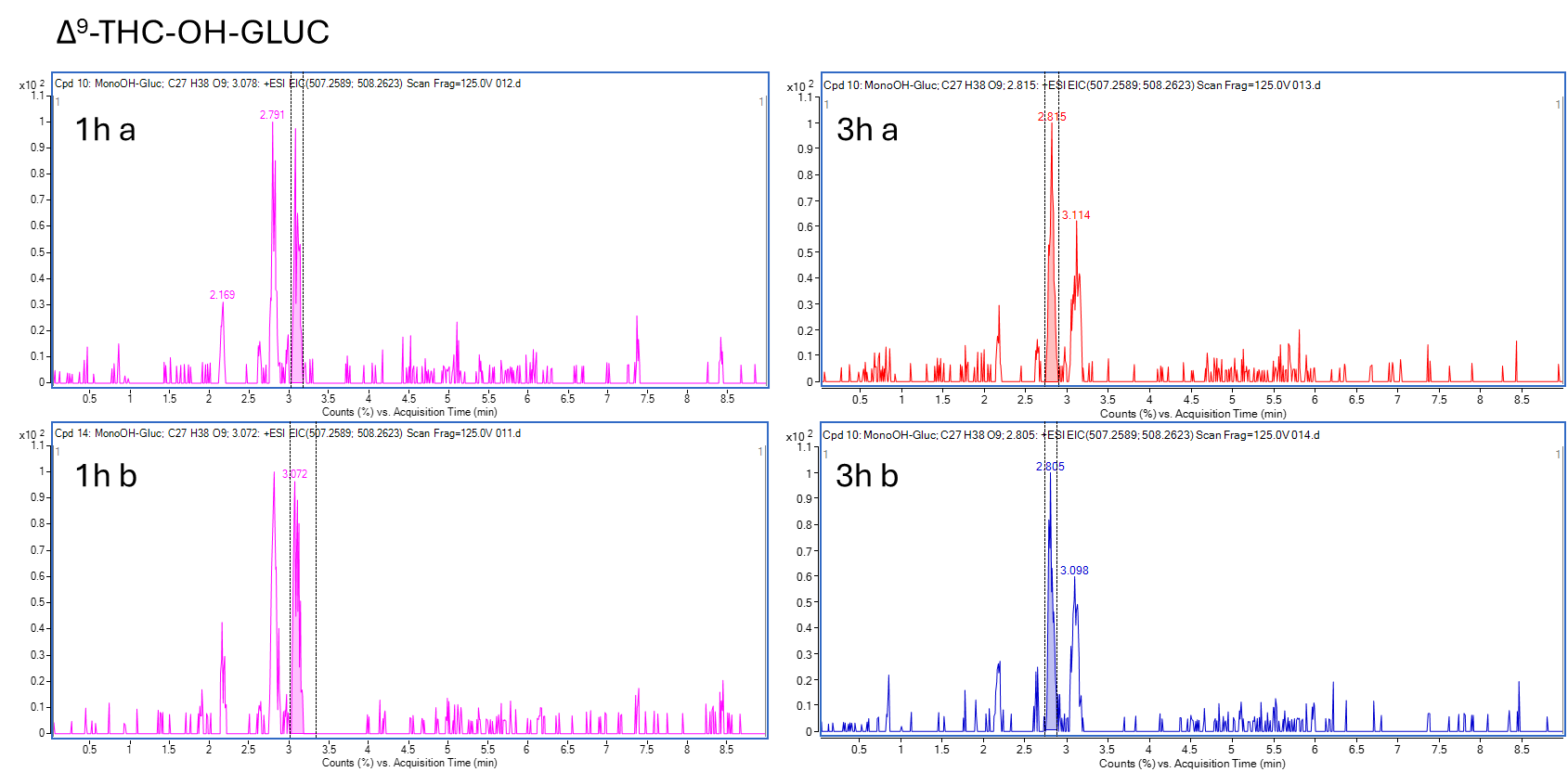


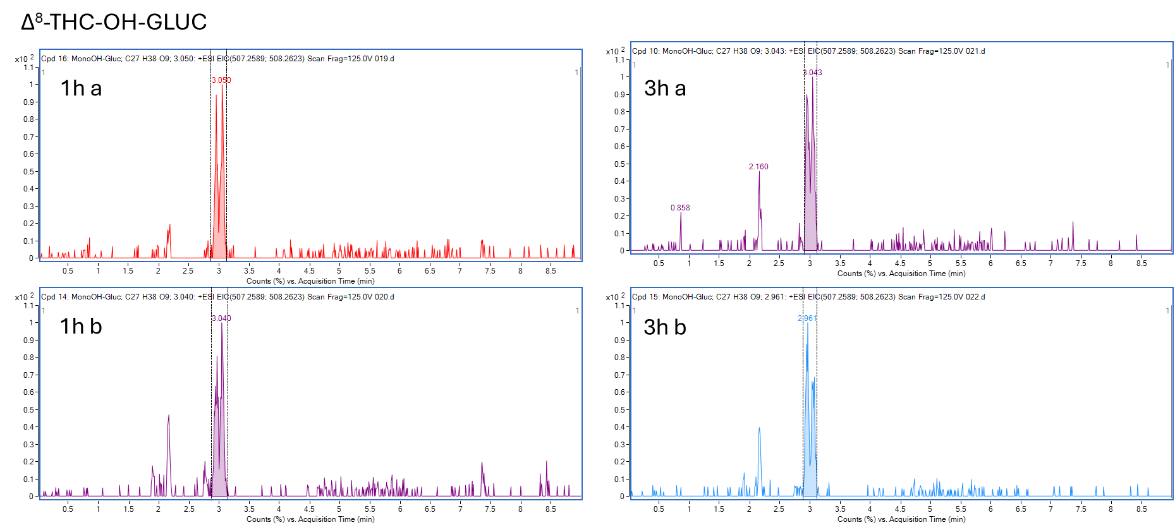


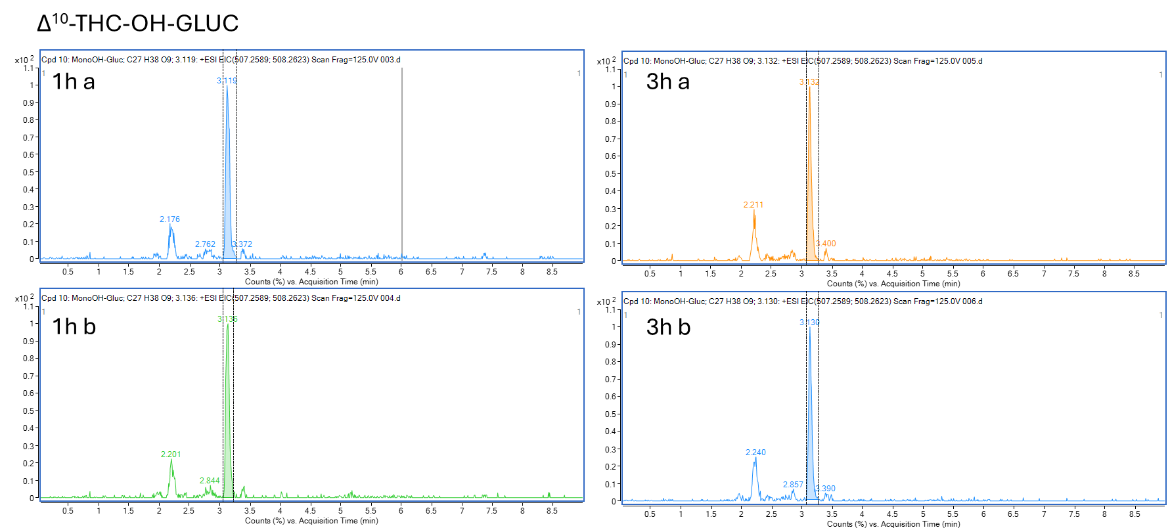


**Chapter 7:** Example chromatograms of authentic urine sample

**Fig. S18:** Chromatograms of an authentic urine sample.

∆^9^-THC (6.2 min)

∆^8^-THC-OH (4.3 min) and ∆^9^-THC-OH (4.5 min)

∆^8^-THC-COOH (4.6 min) and ∆^9^-THC-COOH (4.7 min)
